# Capsule Endoscopy

**DOI:** 10.1155/2012/724084

**Published:** 2012-04-12

**Authors:** Mark E. McAlindon, Reena Sidhu, Marco Pennazio, Cristiano Spada, Martin Keuchel

**Affiliations:** ^1^Department of Gastroenterology, Royal Hallamshire Hospital, Sheffield 510 2JF, UK; ^2^Small-Bowel Disease Section, Department of Medicine, San Giovanni AS Hospital, 10123 Turin, Italy; ^3^Digestive Endoscopy Unit, Catholic University, 000192 Rome, Italy; ^4^Bethesda Krankenhaus Bergedorf, 21029 Hamburg, Germany

Since first described in 2000 [1], capsule endoscopy has established itself as a noninvasive investigative modality of the gastrointestinal tract. It is used as a first-line investigation to image a variety of diseases of the small bowel. These include common conditions, such as coeliac disease, and rare diseases, such as familial adenomatous polyposis, both discussed in detail in this special issue by E. Akin and colleagues.

However, whilst first used to study small bowel disease, these swallowable devices also image the rest of the gastrointestinal tract as peristalsis propels them distally. This is illustrated in the paper by H. Ishiguro et al. in a study using oesophageal capsule to screen for oesophageal varices and referred to in the review of future developments by G. Pan and L. Wang when discussing colon capsule endoscopy.

Whilst avoiding the risks of sedation and intubation associated with conventional endoscopic procedures and the radiation exposure required for many radiological investigations, capsule endoscopy is not entirely without risk. Retention of the capsule behind a stricture is the main concern, particularly if this is a benign disease such as Crohn's or a nonsteroidal anti-inflammatory drug enteropathy which would not necessarily require operative intervention, and the size of this problem is assessed in the large series presented by L. M. Höög et al. These authors also consider the issue of incomplete examinations. This is less widely discussed, but is much more common than capsule retention, may necessitate repeat or alternative investigations, and is an important consideration. The potential for interference with cardiac pacemakers and other devices remains of sufficient concern for manufacturers to consider capsule endoscopy to be contraindicated in the presence of such devices: D. Bandorski et al. provide some reassurance in regard to this matter in their international survey of practice.

G. Pan and L. Wang provide a fascinating insight into what future technological advances might allow us to achieve with these remote devices, which currently just offer an imaging facility but which may develop to allow control of movement, sampling of tissue or fluid and therapy. We should not lose track, however, of the importance of using capsule endoscopy appropriately, safely, and effectively, and R. Sidhu et al. remind us of the need for appropriate training and accreditation to provide excellence of service using devices which are already widely used in routine clinical practice.

*Mark E. McAlindon* *Mark E. McAlindon* *Reena Sidhu* *Reena Sidhu* *Marco Pennazio* *Marco Pennazio* *Cristiano Spada* *Cristiano Spada* *Martin Keuchel* *Martin Keuchel*
